# Advanced Magnetic Resonance Imaging Techniques in Management of Brain Metastases

**DOI:** 10.3389/fonc.2019.00440

**Published:** 2019-06-04

**Authors:** Hatef Mehrabian, Jay Detsky, Hany Soliman, Arjun Sahgal, Greg J. Stanisz

**Affiliations:** ^1^Physical Sciences, Sunnybrook Research Institute, Toronto, ON, Canada; ^2^Radiology and Biomedical Imaging, University of California, San Francisco (UCSF), San Francisco, CA, United States; ^3^Radiation Oncology, Sunnybrook Health Sciences Centre, Toronto, ON, Canada; ^4^Radiation Oncology, University of Toronto, Toronto, ON, Canada; ^5^Department of Neurosurgery and Pediatric Neurosurgery, Medical University, Lublin, Poland

**Keywords:** brain metastases, quantitative MRI, magnetic resonance spectroscopy (MRS), chemical exchange saturation transfer (CEST), diffusion tensor imaging (DTI), magnetization transfer (MT), susceptibility weighted imaging (SWI), relaxometry

## Abstract

Brain metastases are the most common intracranial tumors and occur in 20–40% of all cancer patients. Lung cancer, breast cancer, and melanoma are the most frequent primary cancers to develop brain metastases. Treatment options include surgical resection, whole brain radiotherapy, stereotactic radiosurgery, and systemic treatment such as targeted or immune therapy. Anatomical magnetic resonance imaging (MRI) of the tumor (in particular post-Gadolinium T_1_-weighted and T_2_-weighted FLAIR) provide information about lesion morphology and structure, and are routinely used in clinical practice for both detection and treatment response evaluation for brain metastases. Advanced MRI biomarkers that characterize the cellular, biophysical, micro-structural and metabolic features of tumors have the potential to improve the management of brain metastases from early detection and diagnosis, to evaluating treatment response. Magnetic resonance spectroscopy (MRS), chemical exchange saturation transfer (CEST), quantitative magnetization transfer (qMT), diffusion-based tissue microstructure imaging, trans-membrane water exchange mapping, and magnetic susceptibility weighted imaging (SWI) are advanced MRI techniques that will be reviewed in this article as they pertain to brain metastases.

## Introduction

Brain metastases originate from a large number of primary cancers in the body with breast cancer, lung cancer and melanoma being the most likely to metastasize to the brain (1). Up to 40% of all cancers metastasize to the brain with significant impact on patients' quality of life and survival (2). Surgery is reserved for selected patients with tumors amenable to surgical resection, usually for patients presenting with a solitary, large, symptomatic brain metastasis or when pathological diagnosis is needed. Radiotherapy options include stereotactic radiosurgery (SRS) which precisely delivers high doses of radiation to the tumor—in a single or a few fractions—with the intent of tumor ablation (2, 3); whole brain radiotherapy (WBRT) typically given at doses of 3–4 Gy per fraction over 5–10 treatments sessions; and a combination of SRS and WBRT. Systemic treatment is also being increasingly used to treat brain metastases, especially with new targeted agents and immunotherapy drugs (4–7).

Magnetic resonance imaging (MRI) is widely used in diagnosing brain metastases and differentiating them from other intracranial tumors. MRI is also used in assessing tumor response to treatment, although typically through monitoring changes in the tumor volume alone (8). In clinical practice, two main MRI sequences are routinely acquired: T_1_-weighted acquisition after intravenous injection of gadolinium-based contrast agents (post-Gd T_1w_) which highlights the regions of blood brain barrier disruption and delineates the tumor with relatively high accuracy; and T_2_-weighted fluid-attenuated inversion recovery (T_2w_-FLAIR) acquisition which elucidates areas of vasogenic edema around the tumor. In some clinical protocols, diffusion weighted MRI—usually with three diffusion b-values of 0, 500, and 1,000 [s/mm^2^]—is also acquired in order to provide information about tumor cellularity through measurement of the apparent diffusion coefficient (ADC) (9, 10).

The Response Assessment in Neuro-Oncology—Brain Metastases (RANO-BM) criteria (11) is commonly used in clinical practice and relies on changes in tumor size—which may take weeks or months to occur—to determine response to treatment. Early changes in tumor size do not always correlate with later outcomes (11), which necessitates following patients serially before response can be evaluated reliably. In cases where assessment of local response is uncertain, histopathological evaluation of the tumor via biopsy may be informative; however, it is typically not performed due to associated risks. Also, a needle biopsy often may not be definitive due to sampling error, as a biopsy cannot adequately capture the heterogeneity of the tumor and its response to radiation (12). Typically, serial structural MRI is performed and clinical judgement is exercised to determine the most likely response category: stable disease, progressive disease, or radiation necrosis.

There is an urgent need for advanced imaging biomarkers that provide information about structural, functional, and metabolic changes in the tumor to determine and predict response to treatment sooner and more robust. Such biomarkers should not only characterize tumor morphology and cellularity, but also tumor metabolism, as well as biophysical and microstructural changes (such as apoptosis or cell membrane disintegration) that the cells undergo due to the treatment. These metabolic and microstructural changes generally occur at a much earlier time point than morphological manifestations. For instance, apoptosis begins as early as 4 h post-radiation (13) while volumetric changes may not stabilize until weeks or months post-treatment (8). Such biomarkers have the potential to allow for altering treatment strategies while still within an effective therapeutic time-window.

In addition to diagnosis and treatment response evaluation, MRI is used in monitoring brain metastases after treatment to detect and manage treatment-induced side-effects, as well as detecting tumor recurrence or the development of new metastases. Clinically, the same imaging sequences (post-Gd T_1w_ and T_2w_ FLAIR) are used in follow-up scans which suffer from lack of sensitivity to the underlying metabolic, biophysical and microstructural changes. Therefore, advanced quantitative MRI might enable the much-needed personalization of therapeutic decision making for patients who have undergone treatment for brain metastases.

The current article reviews the advanced quantitative MRI (qMRI) biomarkers that have been applied to brain metastases. Quantitative Imaging section introduces the qMRI techniques that are reviewed in this article and provides background information for understanding the underlying physiological or metabolic processes that each technique probes. In section qMRI in Brain Metastases the applications of each technique in detection and diagnosis of brain metastases, evaluating therapeutic response of the tumor, managing treatment-induced late-effects (e.g., radiation necrosis), and assessing the effects of the treatment on normal brain tissues are discussed. In section Clinical Translation and Limitations clinical translation of these technique and their associated issues as well as their current technical limitations are briefly presented.

## Quantitative Imaging

There exist a large number of quantitative imaging techniques that have been extensively applied to brain metastases. Non-MRI metabolic imaging methods such as fluorodeoxyglucose (FDG) and non-FDG based positron emission tomography (PET) (14), and single-photon emission computed tomography (SPECT) (15), have shown great promise in management of brain metastases. They are however expensive, represent additional imaging (increasing cost and time), and are not in routine clinical use partly due to limited availability.

There is a long history of functional and microstructural MRI-based techniques developed and applied to brain metastases. Dynamic contrast enhanced (DCE)-MRI can be analyzed with a two-compartment Tofts-Kety model to provide quantitative evaluation of vascular permeability and blood flow (16, 17); dynamic susceptibility contrast (DSC)-MRI characterizes tumor perfusion, relative cerebral blood flow (rCBF) and relative cerebral blood volume (rCBV) (18); while ADC measurements calculated from diffusion-weighted MRI reflect tissue cellularity. These functional and microstructural MRI contrasts have also shown promising results in response monitoring and managing treatment side-effects for brain metastases; however, they usually lack the specificity and sensitivity to guide clinical decision-making on their own (19). As MRI has advanced, so has the ability to image with novel qMRI sequences which—if translated to routine clinical practice—have the potential to render biomarkers with the sensitivity and specificity to be clinically useful and will be the focus of the current article.

### Trans-membrane Water Exchange

Each MRI voxel is comprised of cells, microvessels, and extracelluar matrix, etc. Standard MRI measures the “average” signal of water in these tissue compartments, while quantitative MRI tries to disentangle different contributions to the MRI signal. The water molecules constantly move between tissue compartments having different physio-chemical properties in each compartment. The exchange rate of water molecules between intracellular and extracellular compartments, k_IE_, depends on the permeability of the cell membrane as well as the size and shape of the cell (20, 21). This exchange rate is inversely related to the time, τ, that the water molecules spend on average in each compartment (22). This cellular characteristic (k_IE_) changes with treatment; in particular as a result of apoptosis induced by radiotherapy. Apoptosis leads to increased membrane permeability, decreased cell size, and increased irregularity of its shape (21), all of which results in an increase in k_IE_.

The water exchange rate increases in apoptotic cells due to the increased surface-to-volume ratio of the cell either by transformation of the cell into a more irregular shape or decreased overall cell diameter (21, 23), and to a lesser extent due to increased cellular membrane permeability caused by loss of cell membrane integrity (21). In biological tissues, the MR properties (longitudinal, T_1_ and transverse, T_2_ relaxation times) of the intracellular and extracellular compartments cannot be distinguished. However, Gd-based MRI contrast agents do not cross the cell membrane and are purely extracellular. Gd alters both T_1_ and T_2_ of the extracellular compartment in which it is located and also affects the relaxation times of the adjacent compartments indirectly through the exchange of water molecules between compartments (24, 25).

Gd administration disrupts the relaxation equilibrium and makes measuring these relaxation times as well as the trans-membrane water exchange rate constant possible. Trans-membrane water exchange rate, k_IE_, is very sensitive to treatment-induced changes such as apoptosis. In small-scale clinical and pre-clinical studies, k_IE_ has been shown to increase significantly within days after inducing apoptosis (24, 25).

### Susceptibility Weighted Imaging (SWI)

Susceptibility weighted imaging (SWI) exploits the differences in the effective magnetic field in the tissues caused by diamagnetic or paramagnetic substances such as deoxyhemoglobin, iron and calcification (26). SWI signal depends on the deoxy/oxyhemoglobin content in the vasculature which changes due to radiotherapy-induced alterations in tissue microvasculature, specifically caused by the formation of micro-bleeds in the brain (27). Both SWI and the apparent transverse relaxation rate imaging (${\text{R}}_{2}^{*}$) have high sensitivity to hemorrhage and are capable of detecting radiation necrosis. In a pilot study, lower ${\text{R}}_{2}^{*}$ was measured (particularly in the tumor rim) in pseudo-progression compared to progression in patients with GBM (28).

### Magnetic Resonance Spectroscopy (MRS)

Proton magnetic resonance spectroscopy (^1^H-MRS) is sensitive to concentration of tissue metabolites that play crucial role in cancer (29). ^1^H-MRS exploits the fact that in different molecules, there are slight difference in resonance frequency of protons, due to the local magnetic field generated by the local electron cloud surrounding them, a phenomenon called “chemical shift” (30). Molecules detectable with MRS have relatively low molecular weight; are generally able to move between different tissue compartments; and are present in relatively large quantities (>few mM). Some of these metabolites are involved in metabolic pathways of tumors such as involvement of N-acetylaspartate (NAA) in lipogenesis pathways (31); the role of choline (Cho) in the Kennedy pathway [i.e., involvement in genesis of cell membrane phospholipids (32)]; and role of creatine (Cr) in energy metabolism (33), making MRS sensitive to tumor environment (34).

^1^H-MRS data is acquired using either single voxel MRS (SV-MRS) which generates signal from brain sub-regions of approximately a few cubic centimeters; or magnetic resonance spectroscopic imaging (MRSI) which provides higher spatial resolution compared to SV-MRS (35). Neither technique provides sufficient spatial resolution and brain coverage in clinically feasible scan durations, making MRS a region-based acquisition and analysis technique.

**Figure 1:**
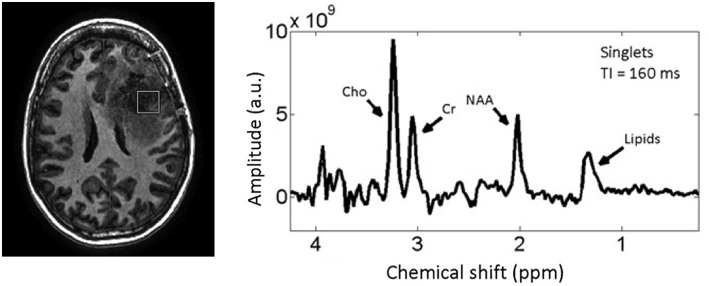
T_1w_ MRI of a patient with a high-grade glioma **(left)** with MRS spectrum **(right)** corresponding to the voxel (white Square) inside the tumor. Reproduced, with permission from Landheer et al. (36).

Figure 1 shows the MRS spectrum of a 2 cm^3^ region of the brain encompassing the tumor in a representative brain cancer patient. The most commonly quantified metabolites with MRS that have been shown—in several small-scale patient studies—to change in tumors and due to treatment are creatine, choline, and NAA (36–42). MRS allows for correlating the concentrations of these sub-cellular molecules with changes in tumor and normal tissue due to treatment (43). However, due to the large voxel sizes MRS is prone to partial volume artifact and its quantitative accuracy is undermined by high tumor heterogeneity within the imaged voxel.

### Micro-Structural MRI

Tissue microstructure and its treatment-induced changes can be probed with diffusion MRI. In addition to the widely-used ADC that is sensitive to cellular density, two more advanced diffusion-based techniques have been used to evaluate intracranial brain tumors: intra-voxel incoherent motion (IVIM) (44–46), and diffusion tensor imaging (DTI) (47).

**Figure 2:**
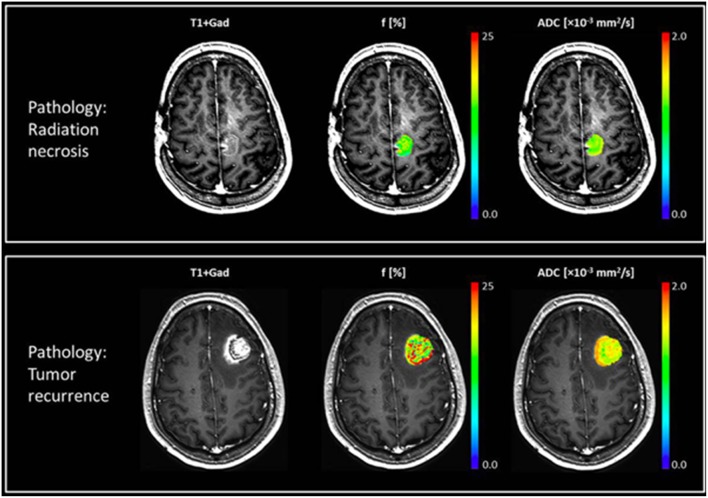
Two patients with brain metastases presenting with enlarging enhancing mass after treatment with SRS. Top row shows a case of radiation necrosis, and bottom row shows a case of recurrent tumor. Post-Gd T_1w_-MRI (left), perfusion fraction *f*-map (middle), and ADC map (right) are shown for both cases, where the patient with radiation necrosis exhibits a uniformly low perfusion fraction while the patient with recurrent tumor has more heterogeneous maps with a higher perfusion fraction. In these two case the ADC values were similar but slightly higher for radiation necrosis. Reproduced, with permission from Detsky et al. (46).

IVIM measures pseudo-diffusion in tissue caused by slow flow of blood through the disoriented capillaries. IVIM model assumes the diffusion MRI signal decay of each voxel is bi-exponential. The fast decaying component represents the motion of the blood in capillaries and the amplitude of this fast decaying component is proportional to microvascular fraction of the voxel. The slow decaying component on the other hand represents the diffusion properties of the tissue (48). The microvascular fraction can also be measured with a simplified IVIM model that focuses on large diffusion b-values (49), and has been used in several pilot studies investigating human brain metastases (45, 46). Figure 2 shows perfusion and ADC maps of microvascular fraction quantification with IVIM for two patients with brain metastases, one having radiation necrosis, and the other with tumor recurrence (46).

DTI on the other hand characterizes the tissue microstructure and water diffusion directionality by performing diffusion sensitization in multiple orientations (50). DTI is sensitive to changes in fiber orientations and also to destruction of white matter tracts caused by radiation or chemotherapy and has been used in several pilot studies to characterize radiation-induced damage to normal brain structures and subsequent cognitive dysfunction (47, 51).

### Quantitative Magnetization Transfer (qMT)

**Figure 3:**
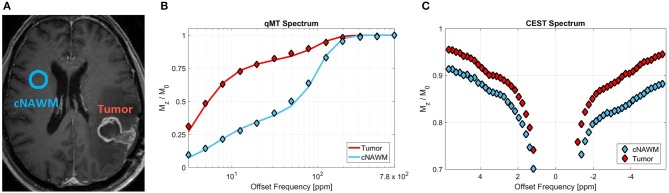
**(A)** Post-Gd T_1w_ MRI of a representative primary brain tumor patient (glioblastoma), showing the tumor and contralateral normal appearing white matter (cNAWM) ROIs. **(B)** The MT spectrums averaged over Tumor and cNAWM, showing the acquired data points as well as the two-pool MT model fit to the data. **(C)** The CEST spectrum averaged over the tumor and cNAWM ROIs. Reproduced, with permission from Mehrabian et al. (53) (License: https://creativecommons.org/licenses/by/4.0/).

Magnetization transfer (MT)-MRI is sensitive to protons associated with large immobile macromolecules that are exchanging with free water protons. Such macromolecules include lipids associated with myelin and cell membranes. Quantitative MT (qMT) data acquisition requires imaging a large range of offset frequencies relative to free water resonance frequency, and a relatively high radiofrequency (RF) power for its magnetization preparation pulse (typically 3–6 μT) (52). This technique characterizes the concentration of the macromolecular protons (i.e., bound proton fraction), the exchange rate between these protons and free water protons, as well as the relaxation rates of the bound and free water pools. All of these characteristics are altered in tumors and also due the the treatment. Figure 3 shows the MT spectrum of a representative patient for tumor and its contra-lateral normal appearing white matter (cNAWM), showing the significant differences between these two tissue types.

qMT mostly represents myelin integrity and to a lesser extent cell membrane integrity (54). qMT has been shown in multiple sclerosis (55, 56) to be sensitive to demyelination resulting from damage to neurons. Smaller MT effect has also been reported in glioblastoma (GBM) tumors and edema as compared to white matter (57). In a pilot study of 20 patients, changes in MT properties of the tumor were found to be more sensitive to treatment-induced changes [such as apoptosis (58)] and reflected these changes much earlier—as early as 2 week into standard chemo-radiation in patients with GBM (53)—than clinically used metrics that rely on morphological changes in the tumor.

### Chemical Exchange Saturation Transfer (CEST)

Chemical exchange saturation transfer (CEST)-MRI is sensitive to concentration and exchange of labile protons including amide (-NH) protons on the backbone of proteins and peptides, amine (-NH_2_) protons on amino acid side-chains, fast exchanging hydroxyl (-OH) protons, as well as intramolecular transfer of magnetization from aliphatic (-CH) protons to labile protons termed as relayed nuclear Overhauser effect (rNOE) (59). These protons can be found in metabolites such as glutamate, lactate, myo-inositol and glucose that play crucial role in brain tumors and their response to therapy (60–62).

CEST relies on the chemical shift between exchanging protons of the metabolites due to their local electron cloud. The dependence of CEST on the exchange as well as the concentration of the proton groups allows for amplification of the CEST effect (using proper imaging and preparation techniques) with several orders of magnitude, making it more sensitive (compared to MRS) to metabolites with very low tissue concentration (62, 63). However, CEST lacks specificity to individual metabolites as it detects chemical groups (e.g., amides, amines, etc.) that are associated with various proteins (64). Certain CEST techniques have recently been developed that are sensitive to chemical groups with a specific range of exchange rates which improve specificity of the measurements (65). The chemical exchange rate in CEST experiments depends on various micro-environmental factors, making CEST a suitable technique for non-invasive measurement of pH (66, 67), which also plays an important role in tumor response to therapy.

Figure 3C shows the CEST spectrum of a brain tumor and its contralateral normal appearing white matter (cNAWM) for a representative patient. The differences in these CEST spectrums arise from the fact that both concentrations and exchange rates of several metabolites—detectable with CEST—change in tumors compared to normal tissue.

Due to relatively high sensitivity to changes in molecular interactions and metabolite concentrations, several pilot studies have shown the potential of CEST in detecting treatment-induced metabolic changes such as radiotherapy induced apoptosis (68, 69). The most commonly used CEST metrics in cancer are amide proton transfer (APT) (70), and magnetization transfer ratio for amide and rNOE (71). These metrics reflect a combination of the CEST effect alongside magnetization transfer and direct water saturation (72). More advanced CEST analysis techniques that better isolate the CEST effect from confounding factors such as Lorentzian decomposition of the spectrum (69), and apparent exchange-dependent relaxation (AREX) (73, 74) have also been developed and applied to cancer.

## qMRI in Brain Metastases

Advanced qMRI techniques have been used in five major aspects of managing patients with brain metastases (all of these investigations were performed on a small number of patients and no large-scale randomized trials have been conducted). Most studies have focused on differentiating brain metastases from other brain tumors such as high and low-grade gliomas (38, 53, 71, 74–81). Assessing tumor response to therapy and attempting to perform such evaluation early after the treatment has been less explored; however, this topic has been gaining significant attention recently (21, 25, 45, 58, 68, 69, 82). Management of treatment-induced late-effects, specifically differentiating radiation necrosis from tumor progression or recurrence, has also been attempted with qMRI techniques. The applications of qMRI that have received little attention are assessing the effects of the tumor and also the treatment on normal brain tissues and their subsequent impact on patients' quality of life (43, 51, 83).

### Detection and Diagnosis of Brain Metastases

Intracranial tumors such as brain metastases, gliomas, and meningiomas may often be differentiated morphologically by their pattern of enhancement on post-Gd scans; however, they sometimes appear similar on anatomical scans, rendering differentiation difficult (84, 85). Although the gold standard for diagnosis is still biopsy, non-invasive methods could be valuable in clinical settings, particularly if a biopsy is not possible.

Significant metabolic, structural, and biophysical differences exist between different brain tumor types that can be exploited by advanced qMRI techniques. N-acetylaspartate (NAA), a major brain neuro-transmitter, is abundant in neurons and its levels correspond to the degree of neuronal destruction (42); High levels of choline (Cho) are associated with increased cell membrane turnover; and increased creatine (Cr) concentration is reported in areas of high energy metabolism (86–88). Increased metabolism and cellularity has been correlated with increased concentration of amide protons and consequently CEST effect (89–91), while a decreased MT effect has been reported in tumors compared to normal brain tissue (53, 57, 77), which could be used in differentiating tumor types.

#### MRS

Brain metastases, similar to GBM, express elevated lipid signal which has been used to differentiate these two tumor types from other brain neoplasms (38). On the other hand, GBM almost always extends beyond the tumor margins as seen on conventional morphological contrast-enhanced MRI (38, 53, 71, 75, 76), while brain metastases are predominantly encapsulated within the enhancing tumor rim (38, 69, 76).

Ishimaru et al. (38), studied 31 patients with high grade glioma and 25 patients brain metastases (primary cancer: 18 lung, 2 breast, 3 colon, 1 ovarian, and 1 malignant fibrous histiocytoma) using single-voxel MRS. They demonstrated lipid signal elevation around 1.3 ppm in majority of patients with GBM and brain metastases. They also showed lipid peak is better detectable in MRS with short echo time, TE (TE = 30 ms) compared to long TE (TE = 136 ms). Caivano et al. (39) has investigated a large cohort of patients involving 32 patients with high-grade glioma, 14 patients with low-grade glioma, and 14 patient with brain metastases (primary cancer: 4 lung, 7 breast, 2 gastric, and 1 melanoma) using multi-voxel 2D MRSI with long TE (TE = 288 ms) to diagnose tumor type. This study concluded that in tumor core the ratios of NAA to creatine (NAA/Cr) and choline to creatine (Cho/Cr) have larger values in brain metastases compared to high and low-grade gliomas (NAA/Cr = 4.43 ± 4.5, 1.68 ± 0.9, 1.04 ± 0.6, and Cho/Cr = 4.88 ± 7.0, 2.7 ± 2.1, 3.4 ± 1.7, for brain metastases, low-grade glioma, and high-grade glioma, respectively). Moreover, in the peri-tumoural edema NAA/Cr and Cho/Cr in brain metastases have larger values compared to high-grade gliomas and smaller values compared to low-grade gliomas (NAA/Cr = 2.53 ± 1.13, 3.73 ± 2.61, 1.49 ± 0.83, and Cho/Cr = 2.72 ± 2.55, 4.62 ± 6.95, 2.49 ± 2.02 for brain metastases, low-grade glioma, and high-grade glioma, respectively), indicating that MRS has the potential to differentiate these three tumor types.

Ishimaru et al. (38) also observed similar trends for NAA/Cr using single-voxel MRS (voxel size ~1.5 cm^3^) with long TE (TE = 136 ms) in 4 brain metastases, 6 patients with low-grade glioma, and 9 patients with high-grade glioma. This study reported statistically significantly higher NAA/Cr ratio for brain metastases compared to gliomas (NAA/Cr = 1.58 ± 0.56, 0.70 ± 0.23, 0.76 ± 0.40 for brain metastases, low-grade glioma, and high-grade glioma, respectively) suggesting its ability to differentiate brain metastases from different types of glioma.

#### Tissue Microstructure

Salice et al. (92) has used a combination of several qMRI techniques including diffusion tensor imaging (DTI), MRS, ADC, and cerebral blood volume (CBV) evaluation, to differentiate benign and malignant brain lesions in 14 patients with similar lesion appearances on anatomical MRI (ring enhancement on post-Gd T_1w_ and surrounding edema on T_2w_ FLAIR). When considering a single parameter, malignant lesions (compared to benign lesions) show lower ADC relative to cNAWM (rADC = ADC/ADC_cNAWM_) on perilesional edema (rADC = 1.4 ± 0.3 vs. 2.1 ± 0.5), and lower fractional anisotropy (FA) of the internal cavity (FA = 0.15 ± 0.09 vs. 0.3 ± 0.02). Malignant lesions also show higher rADC in internal cavity (rADC = 1.8 ± 0.7 vs. 0.6 ± 0.3), and higher FA in perilesional edema (FA = 0.20 ± 0.07 vs. 0.14 ± 0.02) compared to benign lesions. Several combinations of qMRI parameters provided an excellent (>0.9) area under the curve (AUC) of receiver operating characteristic (ROC) curves, with the combination of rADC on the internal cavity, and NAA on the perilesional edema or FA from DTI measurements providing the very high AUC of 0.97, demonstrating their potential in differentiating benign and malignant brain tumors.

#### Magnetization Transfer (MT)

Ainsworth et al. (93) measured magnetization transfer ratio (MTR) and ADC in a mouse model of brain metastases twice a week for 31 days after intracardiac injection of brain-homing breast cancer cell line MDA-MB231-BR. The tumors showed significantly lower MTR and ADC values compared to contralateral normal appearing brain tissue. More importantly, in 24% of cases, they observed significant reduction in both MTR and ADC long before the lesions were detectable on T_2w_ MRI (texture analysis of MTR maps showed 77% sensitivity 2–4 days and 46% sensitivity 5–8 days before lesions were detectable on T_2w_ MRI).

Garcia et al. (77) investigated performance of magnetization transfer ratio (MTR) and qMT parameters in differentiation of brain metastases from other brain tumors in a cohort of 26 patients. They report statistically significantly different MTR and qMT properties (on both the tumor rim and core) for patients with GBM, meningiomas and brain metastases. MTR on the non-contrast-enhancing (CE) region of tumor could only separate brain metastases from meningiomas (MTR [%] = 35.1 ± 0.5, 28.9 ± 1.6, 33.8 ± 1.2 for brain metastases, meningiomas, and GBM, respectively), and MTR on CE region could only separate GBM from meningiomas (MTR [%] = 27.4 ± 1.0, 30.5 ± 1.2, 25.2 ± 0.6 for brain metastases, meningiomas, and GBM, respectively), showing the limited potential of a simple MTR measurement. When considering parameters derived from qMT analysis, macromolecular fraction on the non-CE region of the tumor (M_0b_ [%] = 7.2 ± 0.7, 5.6 ± 0.2, 3.6± 0.7 for brain metastases, meningiomas, and GBM, respectively) and the MT exchange rate on CE region of the tumor (k_f_ [s^−1^] = 0.8 ± 0.1, 1.1 ± 0.1, 0.6 ± 0.0 for brain metastases, meningiomass, and GBM, respectively) could separate all three tumor types.

Furthermore, MT maps show changes in the brain regions that appear unaffected on standard MRI—MT properties are decreased on the ipsilateral and contralateral NAWM of patients compared to healthy controls but are higher than tumor and vasogenic edema—suggesting these advanced techniques provide additional information that could be helpful in the management of these patients (53, 75, 77).

#### CEST

Several studies have used CEST in differentiating brain tumors and also grading them (74, 78–81). However, all of these CEST studies have focused on gliomas or meningiomas, and none included brain metastases. The value of CEST in differentiating brain metastases from other brain tumors remains unexplored.

### Early Treatment Response Evaluation

Determining tumor response to therapy early after the treatment, allows for adjusting strategies for non-responders, while for responders reassures patients and their treating physicians about the treatment effectiveness. Treatment response in clinical practice is currently determined by assessing changes in tumor size on anatomical MRI (11). The earliest clinical time-point for response evaluation in brain metastases is between 4 and 6 weeks after the end of the treatment (11). Radiation-induced effects can mimic tumor growth and may confound response assessment, necessitating longer (3–6 month) follow-up.

Early response evaluation using qMRI can be of great utility, particularly due to its high sensitivity to underlying metabolic, biophysical, and microstructural changes that the treatment induces but are typically too subtle for routine clinically used approaches to detect. Clinically, early identification of non-responders may significantly improve outcomes by allowing for early use of salvage treatments such as surgery or additional radiation.

Radiation-induced changes in cells, such as apoptosis, begin within hours after treatment and preclinical studies have shown the potential of qMRI in detecting radiotherapy-induced changes that are secondary to apoptosis as early as 48 h after treatment (21, 58, 68). Such changes include detection of decreased metabolism through measuring concentration and exchange of amide protons using CEST (68), micro-structural changes in cell membrane integrity through measuring the increased water exchange rate constant between intracellular and extracellular spaces using relaxometry (21), and decreased macromolecular content measured using qMT and increased MTR (mainly due to change in free ware relaxation properties) (58).

#### Perfusion Imaging

Conventional radiotherapy results in an initial increase in perfusion (94, 95); in contrast, stereotactic radiosurgery (SRS) induces a significant reduction in perfusion within a few hours after treatment due to damage to the vascular endothelium (96). These changes can be quantified with perfusion measurement techniques such as IVIM and DCE-MRI (45, 48, 97). Kapadia et al. (45) measured an increase in perfusion index (measured with IVIM) four weeks after treatment (*f* = 0.08 ± 0.02, 0.10 ± 0.03 at baseline and 4 weeks post-SRS, respectively). The study included brain metastases from primary lung (*n* = 8), breast (*n* = 5) and colorectal (*n* = 2) cancers. However, neither perfusion index, which is proportional to tumor blood volume, nor the vascular fraction measured from DCE-MRI were able to differentiate responders from non-responders (45).

#### MRS

Predicting which patients are likely to demonstrate favorable response to radiotherapy (through assessment of tumor aggressiveness), or early prediction of response within a few days after treatment could have a significant clinical impact. Sjobakk et al. (40) measured single voxel proton MRS data (voxel size between 1.0 and 1.5 cm^3^) from 21 patients with brain metastases before treatment (primary cancer: 8 lung, 8 breast, 2 colon, and 3 malignant melanoma). By applying a clustering technique to the MRS spectra between lipids and total choline (between 0.7 and 3.45 ppm), they observed that pre-treatment MRS spectra correlated with 5-month survival of these patients, where patients with higher lipid signal at baseline survived longer. Also, of the four patients that had repeat MRS after treatment, lipid signal decreased after treatment, and among the two patients whose repeat MRS spectrum is shown in the article the patient with larger drop in lipid signal survived longer (16 months vs. 3 months). These results demonstrate the potential of MRS in determining response early after the treatment.

#### CEST

Positive response to treatment is often characterized by decreased tumor metabolism. Metabolism can be probed through characterizing glucose metabolism pathway with FGD-PET. FDG is a widely-used tracer for PET that is preferentially taken up by cancer cells. Using a mouse model, Rivlin et al. (82) showed a similar preferential uptake for 2-Deoxy-D-glucose (2DG). The hydroxyl (-OH) group on 2DG has a strong CEST effect making 2DG-CEST a potential candidate to replace PET without the need for radio-isotopes (82). It could potentially be used in detection and also response monitoring of patients with brain metastases through measuring changes in tumor metabolism.

**Figure 4:**
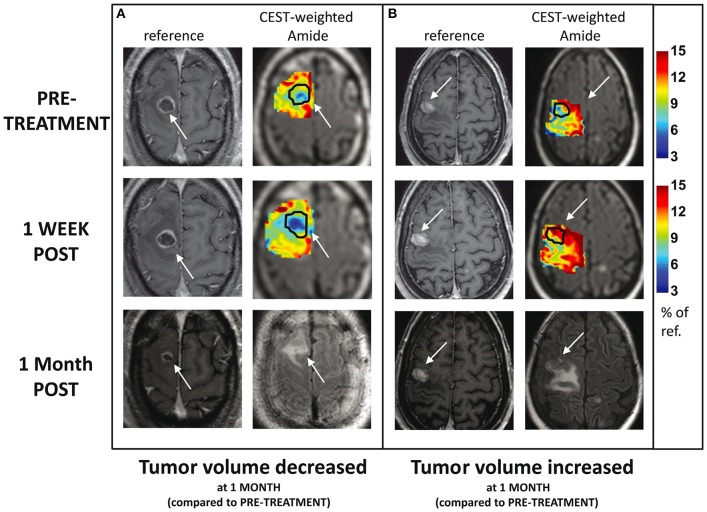
CEST amide MTR maps (tumor and surrounding tissue) for two patients with brain metastases treated with single-dose SRS, at baseline and 1 week after treatment: **(A)** the tumor volume decreased 1 month post-SRS and **(B)** tumor volume increased 1 month post-SRS. The maps are overlaid on T2w FLAIR images. The enhancing tumor region is indicated with arrows and outlined on CEST maps. For comparison, the corresponding slice from the post-Gd T1w MRI is also shown at all three scan time-points. Reproduced, with permission, from Desmond et al. (69).

Desmond et al. (69) applied endogenous CEST-MRI (i.e., without administering a CEST agent) to determine response of patients with brain metastases to single-fraction SRS within 1 week after the treatment (with majority of metastases from primary cancers in lung and breast, and instances of rectum and melanoma). They observe reduced CEST signals after SRS in responders and increased CEST in non-responders (an example for Amide CEST signal is shown in Figure 4). Changes in CEST signals 1-week post treatment (compared to baseline) correlated with the change in tumor volume measured 1 month post-treatment (compared to baseline) with width of NOE peak in the tumor (correlation coefficient, *r* = −0.55, *p* = 0.028) and amplitude of NOE peak on the NAWM (*r* = 0.69, *p* = 0.002) providing the highest correlations (69). Furthermore, the CEST signal amplitude of the NOE peak on cNAWM at baseline scan (before even receiving the treatment) may predict the degree of tumor volume change 1 month post-treatment (compared to baseline) with high negative correlation (*r* = −0.69, *p* = 0.002), indicating its potential in characterizing tumor aggressiveness (69).

#### Relaxometry

Radiotherapy induces microstructural and biophysical changes in the tumor cells undergoing apoptosis which result in increased cell membrane permeability and increased irregularity and shrinkage of the cells (21). The changes in cell membrane integrity as a result of radiotherapy can be probed with the quantification of trans-membrane water exchange rate constant (21). A study of 19 patients with brain metastases treated with SRS (primary cancer: 9 lung, 6 breast, 1 lung and breast, 1 thyroid, 1 endometrium, and 1 rectal), measured a significant increase in trans-membrane water exchange rate constant (due to significant apoptosis) within 1 week after treatment in responders [determined according to RANO-BM criteria (11)], while small changes were measured in non-responders (25).

These studies (25, 69) demonstrate the potential of qMRI in detecting and quantifying radiation-induced metabolic and micro-structural changes in the tumor cells—that precede morphological changes—within days after treatment while adjustment to therapy is still an option. Table 1 summarizes the performance of each technique in evaluating treatment and also their time to detectable response, reported in the studies that were reviews in this section.

**Table 1:** Performance of qMRI techniques in determining response to therapy.

**Biomarker (imaging technique)**	**Response evaluation time**	**Performance**
Perfusion index (IVIM)	4–6 weeks post-treatment	Unable to identify non-responders
Vascular fraction (DCE-MRI)	4–6 weeks post-treatment	Unable to identify non-responders
Spectrum between lipids and choline (MRS)	Baseline	Correlated with 5-month survival
NOE peak width (CEST) NOE peak amplitude (CEST)	1 week post-treatment	Correlated with tumor volume change at 4 weeks
NOE peak amplitude (CEST)	Baseline	Correlated with tumor volume change at 4 weeks
Trans-membrane water exchange (relaxometry)	1 week post-treatment	Correlated with tumor volume change at 4 weeks

### Treatment-Induced Late-Effects

**Figure 5:**
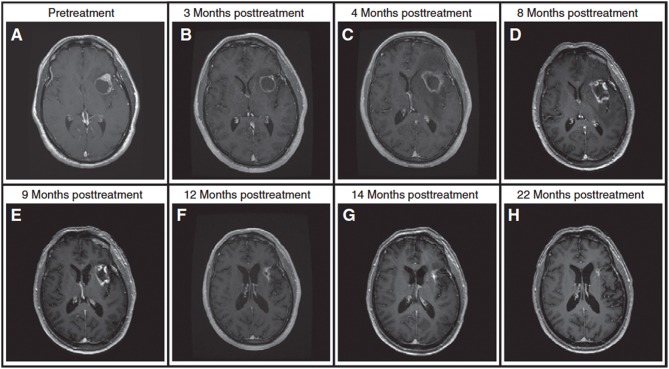
A patient with brain metastasis treated with SRS and presenting with an enlarging enhancing mass after treatment. **(A)** Pre-treatment. **(B)** An enlarging, enhancing mass at the 3-month follow up scan, **(C)** lesion is larger at 4-month follow up. Standard MRI at 3-month and 4-month follow up scans is unable to determine if the lesion is tumor progression or radiation necrosis (lesion is near the language center prohibiting its complete resection). **(D)** A trial of steroids leads to a slight reduction in the enhancing mass but a significant decrease in the surrounding FLAIR at 8-month follow up. **(E)** Continuing steroids leads to further reduction in enhancing lesion at 9-month follow up. **(F–H)** Follow-up MRIs at 12 to 22 months post-treatment scans demonstrate significant decrease in tumor size, rendering a diagnosis of radiation necrosis (the diagnosis is also confirmed with DWI and T_2w_ FLAIR). In two occasions during the period of uncertainly, the patient was admitted to hospital with neurological symptoms. For this patient, it took longer than 9 months to render a diagnosis, demonstrating the challenges faced in clinic in differentiating radiation necrosis from tumor progression. Reproduced, with permission from Mehrabian et al. (72).

Radiotherapy may cause damage in the form of radiation necrosis that may appear several months or even years after the treatment. The likelihood of radiation necrosis increases with radiation dose. Thus, patients treated with high-dose SRS have higher likelihoods of developing radiation necrosis [reported in up to 22% of patients (3, 98)] which can be difficult to manage. It is often impossible to differentiate these radiation-induced changes from tumor progression using standard clinical approaches (98–101); both conditions present with an enlarging enhancing mass in post-Gd T_1w_ MRI and vasogenic edema in T_2w_ FLAIR (3). Figure 5 shows a case where 9 months of follow up imaging was required to determine whether the observed anatomical change represented tumor recurrence or radiation necrosis.

**Figure 6:**
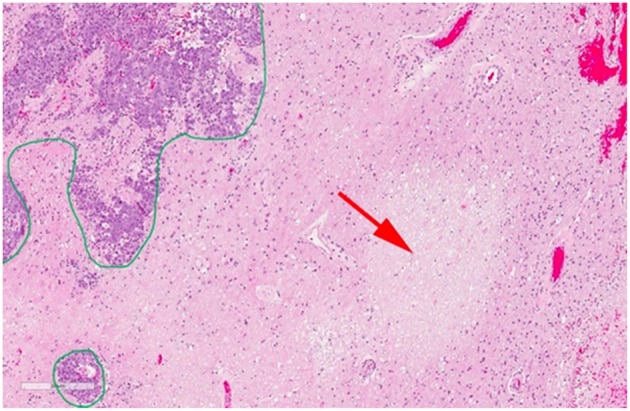
Histopathology of a resected brain metastasis that was previously treated with SRS. The green outline demonstrates residual viable tumor cells while the red arrow shows a region of radiation necrosis. Reproduced, with permission from Detsky et al. (46).

Pathological studies have shown that in most cases there is a mixture of necrosis and residual or recurrent tumor (102) making the diagnosis challenging. Figure 6 shows a case of post-SRS tumor recurrence alongside small areas of radiation necrosis (46). Differentiating between primarily tumor recurrence vs. primarily radiation necrosis is necessary to guide management. Tumor progression is managed with surgery or further radiotherapy while radiation necrosis is managed with observation, steroid therapy, or vascular endothelial growth factor inhibitors such as bevacizumab (8, 103, 104). In the current clinical setting with ineffective means of differentiating tumor progression from radiation necrosis, clinicians have to use their clinical judgment (which may or may not be ultimately correct) or resort to invasive sampling via a biopsy. This leads to significant delays in the appropriate care management [which is palliative in many cases (105)] and can have negative effects on patient's quality of life and survival.

It is hypothesized that radiation necrosis results from radiation damage to the normal white matter, the microvasculature, or a combination of both (106–109). Pre-clinical and clinical studies have shown promising results in differentiating radiation necrosis from tumor progression through characterization of lesion metabolism using CEST and MRS, and probing damage to its macromolecular content using magnetization transfer (MT) (70, 72).

#### MRS

MRS, which evaluates tissue metabolism, has been used extensively in differentiating radiation necrosis from tumor progression in brain metastases. Weybright et al. (37) performed MRS in 29 patients with suspicious lesions after radiotherapy and measured significantly higher ratios of Cho/Cr and Cho/NAA in tumor compared to radiation necrosis. Similar results have been obtained in other studies with Schlemmer et al. (41) reporting that MRS was capable of correct classification of 82% of the lesions in 56 patients with brain tumors (6 metastases, 2 meningiomas, 6 astrocytoma grade I, 6 grade II, 29 grade III, and 9 grade IV) who presented with suspicious lesions and/or clinical symptoms after SRS. Chuang et al. (88) performed a meta-analysis of 13 studies and concluded that in the total of 397 examined lesions, Cho/NAA and Cho/Cr ratios were elevated in tumors (although there was large overlap between values for radiation necrosis and tumor progression reported among different studies). However, in case of the patient shown in Figure 5 (and several other cases), MRS was unsuccessful to render diagnosis (MRS at 4 months post-therapy scan incorrectly suggested the lesion in Figure 5 was tumor progression). These studies show the promise and also some of the limitations of MRS in differentiating radiation necrosis from tumor progression.

#### CEST

Recently several studies have used CEST (in animal models and patients) in differentiating radiation necrosis from tumor progression (70, 72). CEST data was acquired in a study of 16 patients with brain metastases (primary cancer: 6 breast, 2 lung, 3 renal cell carcinoma, 1 melanoma, and 2 non-small cell lung cancer, NSCLC) where 9 patients were later diagnosed—based on clinical guidelines—with radiation necrosis and 7 with tumor progression. Higher CEST signals corresponding to Amide protons (MTR_Amide_ [%] = 8.2 ± 1.0, 12.0 ± 1.9, in radiation necrosis and tumor progression, respectively), and nuclear Overhauser effect (NOE) (MTR_NOE_ [%] = 8.9 ± 0.9, 12.6 ± 1.6, in radiation necrosis and tumor progression, respectively) were measured in progressive tumors compared to radiation necrosis. These CEST metrics differentiated the two lesion types with high accuracy (*p* < 0.0001 for both MTR_NOE_ and MTR_Amide_ (72) in this small-scale clinical study, demonstrating their potential in patient management.

#### SWI

Changes to the micro-vasculature has been studied with susceptibility weighted imaging (SWI) and transverse relaxation rate, ${\text{R}}_{2}^{*}$, mapping. Although these techniques have not been examined in patients with brain metastases, Belliveau et al. (28) used SWI and ${\text{R}}_{2}^{*}$ mapping in nine patients with GBM suspected of having progressive disease (*n* = 5) or pseudo-progression (*n* = 4). They measured higher ${\text{R}}_{2}^{*}$ on both contrast enhancing (CE) and non-CE regions of the lesions in tumor progression compared to pseudo-progression (${\text{R}}_{2}^{*}$ was approximately 60% higher in the CE regions and approximately 14% higher in the non-CE region of the progression cases) (28).

Moreover, in an animal model of radiation necrosis, ${\text{R}}_{2}^{*}$ increased (compared to controls) after radiation in hippocampus—supporting the neuro-inflammatory response to radiotherapy (110)—up to 10 weeks before other radiological signs were detectable (111), demonstrating its high sensitivity to radiation-induced changes in the brain and its promise in differentiating radiation necrosis from tumor progression.

### Tumor Effects on Normal Brain Tissue

qMRI techniques are sensitive to damage to the normal brain structures, in particular neuronal damage. Several studies have observed that even the presence of an intracranial tumor (without any treatment) may lead to alteration or damage to remote brain structures and tissues that appear normal on anatomical imaging. Boorstein et al. (112) studied 15 patients with brain metastases (with non-CNS primary neoplasms) before treatment, to assess the effects of the tumor on the normal appearing brain structures (exclusion criteria was previous cranial radiotherapy or systemic chemotherapy). This study reported no change in the MTR on the cNAWM of the patients; however, they measured significantly lower MTR on the ipsilateral NAWM (outside areas of edema), which may be caused by the destruction of myelin or increased intracellular fluid. Another potential cause of these changes is early formation of new micro-metastases not visible on anatomical MRI.

Similarly, damage to normal brain structures (prior to any treatment) has been reported in other intracranial tumors such as GBM, likely due to their widely invasive and infiltrative nature (75, 113). Such tumor-related normal appearing tissue changes have been detected with DTI (increased fractional anisotropy, FA due to destruction of neurons in cNAWM) (113), qMT (increased direct effect of the free water pool, 1/(R_a_T_2a_) calculated from qMT measurements in cNAWM) (75), and CEST (altered metabolism measured with decrease in Amide and Amine CEST signals in cNAWM) (75). The results of these pilot studies highlight the potential sensitivity of qMRI to tumor-related changes in brain tissues that appear normal on clinical MRI scans.

### Treatment Effects on Normal Brain Tissue

In addition to the effects of the tumor on distant brain tissues, the treatment (radiotherapy and chemotherapy) also significantly impacts the normal (or normal appearing) brain structures. Whole brain radiotherapy (WBRT) plays an important role in the management of patients with multiple brain metastases and can reduce the rate of distant brain failure (114, 115). However, it comes at a cost of decreased cognitive function due to damage to the normal brain structures and results in a detriment to the patients' quality of life, particularly those with extended survival (51, 106). Considering the palliative nature of the treatment for brain metastases, sparing normal brain function and avoiding impairment to patients' quality of life is of utmost importance in their management.

**Figure 7:**
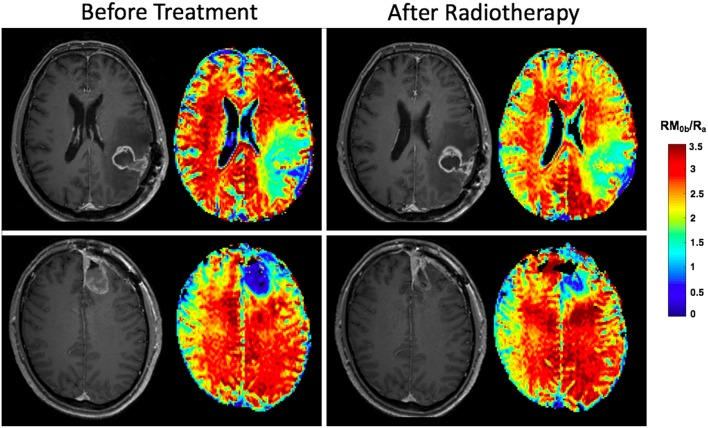
Effects of radiotherapy plus Temozolomide on normal tissue of two patients with GBM. Both patients received IMRT at a dose of 2Gy/day. After the first 10 fractions, the white matter on the contralateral side of the brain on post-Gd T_1w_-MRI appears normal before and after treatment for both patients. The parametric maps show the amount of magnetization transfer (MT), RM_0b_/R_a_ which quantifies white matter integrity. The patient in the top row experiences significant decrease in amount of MT showing significant white matter damage, while for the patient in the bottom row, the amount of MT has not changed showing the patient's resistance to radiation.

Once the tumor is treated with radiation, a decrease in qMT parameters (such as amount of magnetization transfer, RM_0b_/R_a_) is observed even after a few radiotherapy sessions due to disruption of the white matter integrity. Unpublished data in Figure 7 shows effects of 10 treatment session with 2Gy/day on two patients with GBM, showing the different response of the normal brain tissue of the patients to radiotherapy plus chemotherapy where one patient experiences significant change in qMT parameter and the other patient experiencing no change.

Pospisil et al. (43) investigated 18 patients with brain metastases (primary cancer: 1 lung, 5 breast, 5 renal cell carcinoma, 3 NSCLC, 2 gastrointestinal, 1 gynecological, and 1 other cancer type) undergoing WBRT with MRS (before and 4 months after WBRT). This study reported significant decrease in hippocampus NAA after treatment (a marker of neuronal loss), which was accompanied by a decrease in the patients' quality of life. The loss of hippocampal NAA has also been correlated with cognitive decline after WBRT (83).

DTI has also been used in assessing radiotherapy effects on normal brain microstructure. Chapman et al. (51) acquired DTI data before radiotherapy, during radiotherapy (3 weeks and 6 weeks after start of radiotherapy), and after radiotherapy (10, 30, and 78 weeks after start of radiotherapy) in 10 brain cancer patients and performed neurocognitive functional tests as well as measuring quality of life metrics. They reported decreased longitudinal diffusivity (significant at 6-week scan compared to pre-treatment) and increased perpendicular diffusivity (significant at 10-week scan compared to pre-treatment)—both indicators of neuronal integrity destruction. They also observed positive correlation in percent change (compared to pre-treatment) of longitudinal diffusivity at 6-week scan, with its change at 30-week scan (correlation coefficient, *r* = 0.70, *p* < 0.05) indicating early changes in this parameter may be able to predict its later changes.

Chapman et al. (51) also reported dose dependence of the DTI changes; particularly for perpendicular diffusivity which at 3-week scan correlated with radiation dose (*r* = 0.49, *p* < 0.05). In addition, they reported linear correlation between longitudinal diffusivity post-radiotherapy (30-week scan), and verbal recall scores of the patients (*r* = 0.73, *p* < 0.02); and observed that longitudinal diffusivity during radiotherapy (3-week and 6-week scans) could predicts post-radiotherapy verbal recall scores (*p* < 0.05) (51). These studies, although performed in small number of patients, demonstrate the potential of qMRI techniques in characterizing treatment-induced changes in normal appearing brain structures, which may be useful in patient management.

## Clinical Translation and Limitations

Brain metastases originate from multitude of primary cancers with breast cancer, lung cancer, and melanoma being the most frequent cancers to metastasize to the brain. Brain metastases carry several characteristics of the primary tumor, for instance microvasculature of the brain metastases is different from that of the normal brain and mimics the microvasculature of the original tumor (i.e., lack of neuro-vascular unit components that leads to uniformly increased vasogenic edema) (116). The similarities between brain metastases and their primary tumors necessitates investigating the qMRI markers for each primary site, since what is true for metastasis from one tumor might not necessarily be true for metastasis from another primary site. This issue was not considered in any of the studies that were reviewed here. These studies were all pilot studies and were evaluating the potential of new MRI techniques in management of brain metastases, with the goal of developing new biomarkers. Thus, they enrolled as many patients as possible with metastases from different primary sites. Clinical studies with focus on metastases from one primary site are needed to determine if the primary site has an impact on the performance of these biomarkers.

qMRI techniques have the potential to assist physicians in managing patients with brain metastases. However, the evaluation of these techniques has been limited to small, single-center studies due to limited availability of the imaging sequences, as well as lack of expertise and standardization for widespread clinical use. All the studies that were reviewed here were conduct on a small number of patients, many of them at one institution and were conducted by research teams that either developed the technique or are experts in applying them. Large multi-center clinical trials are needed to fully assess the potential of these biomarkers and their clinical utility. Standardization of the techniques and development of analysis tools that could be used by users in a clinical setting is crucial for their clinical translation.

Incorporation of advanced qMRI techniques in clinical practice results in longer MRI scans (usually 60–90 min). Given the general health state of brain metastasis patients, they may not be able to easily tolerate the requirement for staying still in the MRI scanner for long scans. In the authors experience around 60-min scans were well-tolerated by the patients, however, attrition rate of 20–30% was reported in patients that attended the first scan but did not complete the study (71). Optimization of the scan protocols and establishing the benefit of the added MRI sequences to the patient care might increase patient participation rate. Finally, many of these techniques such as CEST, qMT, and MRS require very long scan times and provide reduced brain coverage, limiting their value in clinical decision making. Technological developments are needed to accelerate these techniques to allow for better coverage without losing specificity and sensitivity. Many of these technical issues have not been studied for the introduced qMRI techniques and need to be investigated and addressed before any clinical translation.

## Author Contributions

HM performed the literature review and prepared the manuscript. JD, HS, AS, and GJS contributed equally to the preparation of the manuscript.

### Conflict of Interest Statement

AS declares past educational seminars with Elekta AB, Accuray Inc., and Varian medical systems; research grant with Elekta AB; and travel accommodations/expenses by Elekta and Varian. AS also belongs to the Elekta MR Linac Research Consortium. The remaining authors declare that the research was conducted in the absence of any commercial or financial relationships that could be construed as a potential conflict of interest.
